# Psychological symptoms and their correlates in pneumoconiosis patients: a bilingual scoping review

**DOI:** 10.3389/fpubh.2025.1703091

**Published:** 2025-11-13

**Authors:** Xi-Ping Wang, Runxuan Li, Kim-Pong Tam

**Affiliations:** 1 Division of Social Science, The Hong Kong University of Science and Technology, Hong Kong, Hong Kong SAR, China; 2 Institute for Management and Innovation (IMI), University of Toronto Mississauga, Toronto, ON, Canada

**Keywords:** pneumoconiosis, mental health, occupational health, psychological symptoms, pneumoconiosis patients

## Abstract

**Purpose:**

Pneumoconiosis remains a global occupational and public health problem. This scoping review is aimed to identify the psychological symptoms and their correlates among pneumoconiosis patients.

**Methods:**

We conducted a bilingual scoping review following the PRISMA-ScR guidelines. Studies published between 2000 and 2025 were identified through searches of English-language (PubMed, Web of Science, Scopus, ScienceDirect) and Chinese-language (CNKI, CBM, Wanfang, VIP) databases, using English and Chinese descriptors related to pneumoconiosis and psychological symptoms. The search was conducted from June 28 to July 5, 2025. Peer-reviewed empirical studies that reported quantitative psychological outcomes among patients diagnosed with pneumoconiosis were included.

**Results:**

A total of 55 studies were included in this review: 11 in English and 44 in Chinese. Considerable variability in reported prevalence was observed, primarily driven by differences in the instruments used and the cutoff criteria. For depression, with different diagnostic tools and criteria, Chinese studies reported prevalence ranging from 16.3% to 87.22%, and English studies reported prevalence ranging from 75.2% to 86.1%. For anxiety, again with varying assessment tools and criteria, Chinese studies reported rates from 9.5% to 61.97%, and English studies reported rates from 75% to 99.1%. Some studies also reported somatisation, obsessive-compulsive symptoms, and sleep disturbances. Key correlates associated with these symptoms included biological, social, and psychological factors.

**Conclusion:**

This review synthesises evidence on the high prevalence of psychological symptoms among pneumoconiosis patients across diverse regions, which are associated with multifaceted factors. The scarcity of intervention studies, combined with methodological heterogeneity in existing research, underscores the urgent need for standardized assessment tools and the development of context-sensitive, biopsychosocial care models for this population.

## 1 Introduction

Pneumoconiosis is a category of respiratory diseases that includes asbestosis, silicosis, coal worker’s pneumoconiosis, talc pneumoconiosis, kaolin pneumoconiosis, siderosis of the lung, and other rarer forms, all resulting from sustained inhalation of mineral dust particles during occupational exposure (1). Despite improved occupational safety regulations over the years, pneumoconiosis remains a serious global public health challenge nowadays (2). Annual deaths have exceeded 21,000 each year since 2015, according to estimation (3). The global annual incidence of pneumoconiosis increased by 61.5% between 1990 and 2019, rising from 123,271 cases to 199,125 cases (4).

Research shows that individuals with chronic diseases in general experience poorer mental health more frequently than those without chronic conditions (5). Social, material, and relational problems increase mental health risks. Specific stressors, such as financial strain, housing instability, intimate relationship difficulties, and sexual concerns, occur more often among chronic disease populations (6). Considering that most pneumoconiosis forms are chronic conditions, the mental well-being of pneumoconiosis patients demands attention from public health and occupational health systems. Indeed, existing studies have revealed higher mental health risks among pneumoconiosis patients (7, 8). However, to date, no review has systematically synthesized findings across both publications in the Chinese and English languages. This knowledge gap prevents a complete understanding of the symptom patterns and management needs of pneumoconiosis patients. In response to this situation, the present scoping review aims to map the existing body of evidence across diverse study designs and across the two languages.

Current research on the mental health of pneumoconiosis patients rarely considers the insights from both studies published in English and Chinese. This oversight persists even though the disease poses a severe health challenge in China. Pneumoconiosis is the most prevalent occupational disease in China. Data from China’s National Health Commission reveals that pneumoconiosis accounts for 90% of all occupational diseases (9). In 2019, China reported the highest incidence of the disease among all countries (136,755 cases), followed by India (11,670 cases) and the United States (10,014 cases) (5). Furthermore, China accounted for 68.7% of new pneumoconiosis cases, 44.3% of related deaths, and 66.2% of disability-adjusted life years (DALYs) globally (10). While this review adopts a global perspective, we expected that a substantial part of the evidence would come from China.

According to the data above, China accounts for a significant portion of the global pneumoconiosis cases (5, 10). Consequently, excluding Chinese-language literature would have created a significant evidence gap. Such an exclusion could have biased the overall findings. Therefore, we believe there is a need to consider evidence from both English- and Chinese-language studies. This approach allows our study to present a more comprehensive view of existing research.

In summary, the present scoping review aims to synthesize existing evidence by addressing the following questions:

1. What is the reported prevalence of psychological symptoms (e.g., anxiety, depression) among pneumoconiosis patients in the Chinese and English literature?
2. What biological, psychological, and social factors are associated with these symptoms?
3. What evidence exists regarding the effectiveness of interventions for these symptoms?

By mapping the existing body of research, we attempted to identify crucial directions for future research and strategic priorities for policy decisions.

## 2 Method

This scoping review follows the procedures recommended by PRISMA Extension for Scoping Reviews (PRISMA-ScR) (11). A preregistration of the review was submitted to Open Science Framework.^1^ Our approach follows the general scope of inquiry (PCC: Population = pneumoconiosis patients; Concept = psychological symptoms and their correlates; Context = global research literature). Unlike systematic reviews, PRISMA-ScR does not require scoping reviews to include a formal quality assessment (risk of bias appraisal) of the included studies or a statistical meta-analysis (11). The primary goal of a scoping review is to map the scope and nature of the available evidence and to pinpoint gaps in the research (11).

We conducted a systematic search of these databases for English literature: APA Databases (PsycArticles and PsycINFO), ProQuest, PubMed, ScienceDirect, Scopus, and Web of Science. For Chinese-language literature, we used the following databases: Chinese Academic Journal (CNKI), Chinese Biomedical Literature Database (CBM), VIP database, and Wanfang data. We selected databases to ensure comprehensive global and regional literature coverage. English-language databases (including APA Databases for psychology, PubMed for biomedicine, and Scopus/Web of Science for multidisciplinary content) provide international evidence. Due to China’s high pneumoconiosis burden, Chinese medical databases (CNKI, CBM, Wanfang, VIP) were essential.

We used consistent Boolean logic across all databases: (“pneumoconiosis”) AND (“anxiety” OR “depression” OR “mental health” OR “psychological distress”), and then applied filters for time range, language, and human subjects in some databases. However, some databases do not support excluding animal studies, selecting language, or displaying only peer-reviewed articles when performing searches. When searching Chinese databases, we applied semantically equivalent adjustments to the search logic using simplified Chinese terms with identical meanings. Two native Chinese-speaking researchers independently translated core English terms (e.g., “anxiety,” “depression”) using ICD-11 and DSM-5 diagnostic criteria. Separate translators performed back-translation to verify semantic equivalence. The final Chinese terms (e.g., 尘肺病 for pneumoconiosis, 焦虑 for anxiety) underwent validation through test searches across all Chinese databases. This procedure confirmed retrieval accuracy before conducting the complete literature search. Our searches covered title, abstract, and keywords. We provide a supplementary document presenting the specific search query used for each database.

All databases were searched from June 28 to July 5, 2025, covering publications from 2000 to 2025. Over the past few decades, the global incidence of pneumoconiosis has been rising (5). We selected the 2000–2025 timeframe for this review to zoom in on emerging research developments since the onset of the 21st century.

Two reviewers (including the first author and an independent reviewer who has a bachelor’s degree) independently screened the results from the searches in two phases:

1. Title/Abstract Screening: Using Rayyan software, two reviewers assessed eligibility through reading titles and abstracts.
2. Full-Text Review: The same two reviewers evaluated potentially eligible studies based on eligibility criteria as checklists, resolving disagreements through consensus discussion. If a consensus could not be reached, a third senior researcher would arbitrate.

Table 1 shows the eligibility criteria for inclusion and exclusion.

**Table 1:** Eligibility criteria.

Inclusion criteria	Exclusion criteria
Population of diagnosed pneumoconiosis patients (all stages/types) Quantitative reporting of psychological Symptoms (e.g., SAS/SDS/SCL-90 scores) or predictor analyses	Non-pneumoconiosis populations (e.g., dust-exposed workers without diagnosis) Studies reporting only physiological outcomes (e.g., lung function)
Peer-reviewed articles	Not peer-reviewed
Written in Chinese or English (2000–2025)	Non-empirical publications (editorials, commentaries) Not written in Chinese or English Other Animals studies (non-human)

We excluded grey literature (e.g., reports and theses) and non-empirical publications (e.g., editorials, commentaries, and reviews). We excluded grey literature due to its unclear and debated definitional criteria. Moreover, we focused only on peer-reviewed studies to ensure scientific rigor in the evidence to be reviewed.

We excluded qualitative studies; this decision aligned with the specified objectives of our review, which are to map the prevalence of psychological symptoms and identify biological, psychological, and social factors reported in the empirical literature. To meet these objectives, we had to rely on quantitative evidence. In the English-language literature search, we identified 128 articles from the database searches. We initially removed 65 duplicates. Subsequently, based on a review of titles and abstracts, we excluded 48 articles that were not related to the topic, did not report quantitative psychological symptoms, were animal studies, were not written in English, or were not peer-reviewed. Then, we conducted a full-text eligibility assessment of the remaining 15 articles and thereby excluded four additional articles that were unrelated to the topic or not peer-reviewed. In the end, we included 11 articles in the present review.

In the Chinese literature search, we identified 302 articles from the database searches. We initially removed 116 duplicates and then excluded 137 articles that were not related to the topic or did not report quantitative psychological symptoms. Thereafter, we conducted a full-text eligibility assessment on the remaining 49 articles and decided to exclude five articles that were not related to the topic, lacked transparent reporting of the data collection process, or did not report quantitative results. Finally, we included 44 articles in the present review.

**Figure 1:**
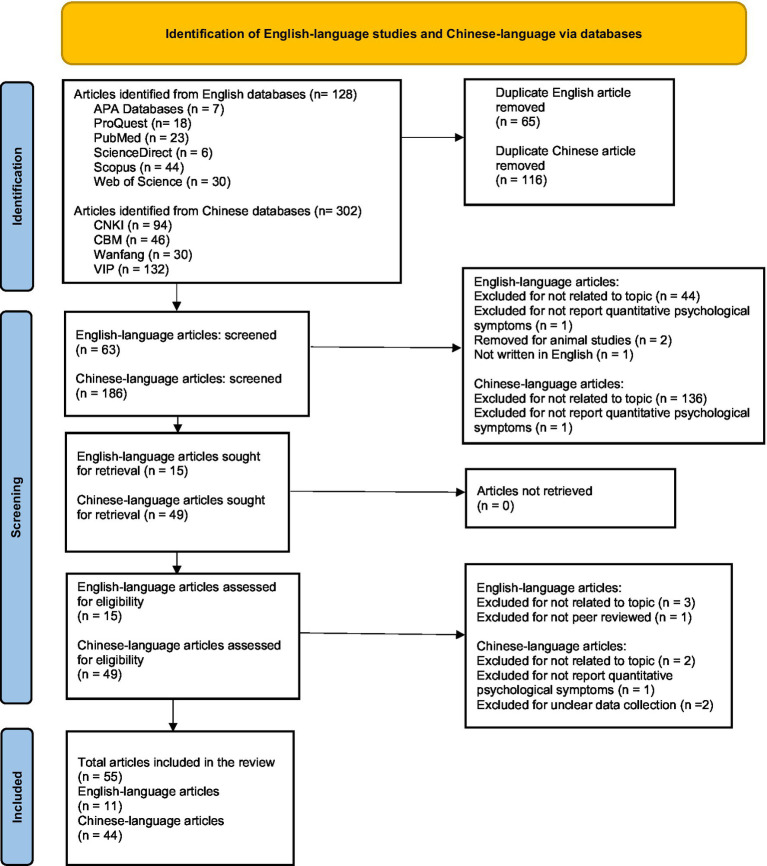
PRISMA 2020 flow diagram for screening.

Figure 1 shows the screening process.

## 3 Results

This analysis included 55 studies, 11 in English and 44 in Chinese, spanning the years 2003–2024. Table 2 details the English-language studies. Publication of these studies occurred mainly after 2010, with over half (six out of 11 studies) appearing in 2020 or later. This pattern suggests a recent increase in international interest. These studies came from specific geographic locations: one from South Korea, one from Japan, one from Romania, one from Taiwan, China, four from Hong Kong SAR, China, and three from mainland China. Table 3 details the Chinese-language studies. All these studies focused exclusively on mainland China. The regions investigated in China were diverse, including Chongqing, Ningxia, Shandong, and Guangdong. Over 80% of these studies (36 out of 44) were published in 2010 or later, with most (28) concentrated in the most recent decade (i.e., 2014–2024).

**Table 2:** English-language studies.

Study	Location	Method/sample size	Psychological symptoms (prevalence/score)	Correlates
Cho and Cho (19)	South Korea	Cross-sectional (*n* = 161 older pneumoconiosis patients)	Depression: Mean GDS-15 score = 8.90 ± 3.26 (75.2% scored ≥7); Death anxiety: Mean score: 94.04 ± 13.38 (Highest sub-score): “Dying process of oneself” (3.07 ± 0.42).	QoL Correlates (SGRQ-Higher score = worse QoL): ↓QoL associated with: No spouse, Lower education (vs. uneducated), No religion, Lower monthly income, Time since diagnosis, Longer hospitalization. Mediation: Death Anxiety mediates the relationship between Depression and QoL
Han et al. (20)	Shenyang, China	Cross-sectional (*n* = 324 silicosis inpatients)	Anxiety: Prevalence 99.1% (HADS-A ≥ 8); Mean score: 12.9 ± 1.9; Depression: Prevalence 86.1% (HADS-D ≥ 8) Mean score: 10.5 ± 1.9; Social Support: Mean MSPSS 55.3 ± 10.3 (low level)	Social Support: Significant negative association with anxiety and depression 2. Education Level: Positive association with anxiety 3. Age: Negative association with depression 4. Gender: Females reported lower social support and higher depression
Kawaji et al. (30)	Japan	Cross-sectional (*n* = 185: 171 men, 14 women)	Depression: Median SDS score = 49 (IQR 44–52) (indicating clinically significant depression); Outcome Expectations (OE): Median score = 14 (IQR 10–18).	Dyspnea (mMRC): Significant negative association with physical activity (PA) (*β* = −0.20, *p* = 0.008). Directly impacted PA, OE, stage of change, QOL, life space (LSA), and depression. Outcome Expectations (OE): Significant positive association with PA (*β* = 0.26, *p* = 0.001). Directly impacted PA; mediated by self-efficacy and decisional balance.
Lee et al. (7)	Taiwan, China	Retrospective cohort study (*n* = 16,795 pneumoconiosis patients vs. 67,180 without pneumoconiosis)	Depression Incidence: 10.07 vs. 5.99 per 1,000 person-years (pneumoconiosis vs. control) Adjusted HR = 1.84 (95% CI = 1.70–1.99; *p* < 0.001)	Disease Severity (ED visits): Dose-dependent risk escalation (*p* < 0.001): 1–2 visits/year: aHR = 1.34 (1.13–1.59) ≥2 visits/year: aHR = 2.31 (1.94–2.74). Age: Strongest association in ≥80 years: aHR = 2.25 (1.83–2.75). Sex: Higher risk in women: aHR = 1.45 (95% CI = 1.32–1.58) vs. men. Comorbidities: Hypertension (aHR = 1.43), ischemic heart disease (aHR = 1.28), cerebrovascular disease (aHR = 1.25), asthma/COPD (aHR = 1.31), chronic liver disease (aHR = 1.35). Socioeconomic: Higher income (≥40,000 NTD): aHR = 1.14 (1.03–1.26)* Low urbanization: Protective trend (aHR = 0.92).
Liu et al. (32)	Shenyang, China	Cross-sectional (*n* = 208)	Depression: Median GDS-15 score = 5 (range 1–15); Social Support (SSRS): Median = 35 (range = 20–52).	Activity Impairment: Significantly associated with lower SF-36 PCS (OR = 0.555, 95% CI: 0.319–0.967) and MCS scores (OR = 0.421, 95% CI: 0.212–0.834). Depression: Significantly associated with lower SF-36 MCS (OR = 0.073, 95% CI: 0.036–0.146). Financial Compensation: Highlighted as a potential buffer for stress and social support maintenance (discussion section). Age: Patients ≥65 years had significantly worse physical function (PF, RP, BP, GH, SF, RE) vs. controls.
Postolache et al. (22)	Romania	Cross-sectional (*n* = 16 pneumoconiosis patients)	Anxiety and Depression Prevalence: 75% (12/16 patients, HADS ≥8) Mean HADS: 13.5 ± 6.87 Quality of Life (QoL): SGRQ-Total: 53.48 ± 15 SGRQ-Activity (most affected domain): 72.39 ± 19.9.	Dyspnea severity: Strong correlation with HADS (*r* = 0.512, *p* < 0.01); Pulmonary function: Higher FEV1% predicted (49.97 ± 18.56) vs. asthma/COPD; Disease type: Lower HADS in pneumoconiosis (13.5) vs. COPD (16.05); Functional impairment: Activity limitation (SGRQ-Activity) most severe across all OLD.
Tang et al. (26)	Hong Kong, China	Cross-sectional (*n* = 297 male pneumoconiosis patients)	Depression: Mean GDS score 7.3 ± 3.8.	GDS score: Strongest predictor of SGRQ scores (*β* = 0.39 for total score, *p* < 0.0005) FVC% predicted: Significant negative association with SGRQ scores (*β* = −0.28 for total score, *p* < 0.0005) Comorbidity count: Positive association with SGRQ impact (*β* = 0.12, *p* < 0.05) and total scores (*β* = 0.15, *p* < 0.05) Age: Associated with SGRQ activity score (*β* = 0.18, *p* < 0.005).
Tang et al. (40)	Hong Kong, China	Cross-sectional (*n* = 201 older Chinese patients with pneumoconiosis)	Depressive disorders: Prevalence 9.5% (DSM-IV diagnosis via SCID). Subtypes: Minor depression (4.5%), Major depression (3.57%), Dysthymia (1.5%). Geriatric Depression Scale (GDS) Score: 7.1 ± 3.9.	IADL Score: OR = 1.17 (95% CI: 1.07–1.29; *p* = 0.001) Higher score = poorer function = ↑ depression risk LSNS Score: OR = 0.94 (95% CI: 0.90–0.99; *p* = 0.019) Lower score = poorer social support = ↑ depression risk Univariate association: Number of comorbidities (*p* = 0.002).
Tang et al. (52) Tang et al. (36) Wang et al. (31)	Hong Kong, China Hong Kong, China China	Cross-sectional (*n* = 201 older patients with pneumoconiosis) Cross-sectional (*n* = 112 patients and their caregivers) Cross-sectional (*n* = 121 silicosis patients vs. 110 controls)	Depression: Prevalence: 9.5% (DSM-IV diagnoses: major/minor depression or dysthymia); Screening Performance: Geriatric Depression Scale (GDS-15) mean score: 7.1 ± 3.9 Optimal cut-off: 9/10 (sensitivity 84%, specificity 75%, PPV 0.26, NPV 0.98, AUC 0.90). Caregiving Burden: Mean CBS 44.3 ± 10.3 (moderate). Caregiver Depression: Mean GDS 5.7 ± 3.7 (low level). Quality of Life: PCS 54.4 ± 10.0; MCS 51.8 ± 10.1. Depression: Prevalence 27.3% (BDI ≥ 14) in the silicosis group vs. 7.3% in controls.	N/A Caregiving Burden (CBS): ↓ Patient daily functioning (*β* = −0.345), ↑ Caregiver depressive symptoms (*β* = 0.509), ↓ Availability of family support (*β* = 0.240); Physical QOL (PCS): ↑ Patient severity of coexisting diseases (*β* = −0.179), ↑ Caregiver depressive symptoms (*β* = −0.521); Mental QOL (MCS): ↑ Patient depressive symptoms (*β* = −0.155), ↑ Caregiver depressive symptoms (*β* = −0.633), ↑ Patient severity of dyspnea (*β* = −0.183). In Silicosis Patients (Adjusted OR): Severe symptoms (SGRQ): OR = 3.8 (95% CI 1.1–13.2) Severe impaired physical function (SGRQ): OR = 3.4 (95% CI 1.0–11.2)FEV1 < 50% predicted: OR = 3.6 (95% CI 1.3–10.2) FVC% predicted < mean (58.3%): OR = 3.2 (95% CI 1.1–9.5).

**Table 3:** Chinese-language studies.

Study	Location	Method/sample size	Psychological symptoms (prevalence/score)	Correlates
Cao et al. (12)	Ningxia, China	Case–control (*n* = 160 CWP patients vs. 110 controls)	Depression: Prevalence 45.6% (HAMD ≥17) in the CWP group vs. 5.5% in controls. Anxiety: Prevalence 40.0% (HAMA ≥14) in CWP group vs. 7.3% in controls.	In CWP Patients: Association with CWP Stage: No significant difference in HAMD or HAMA scores across stages I, II, and III (*p* > 0.05).
Chen et al. (43)	Zhengzhou, China	Cross-sectional (*n* = 196 male pneumoconiosis patients)	SCL-90: Somatization: Mean 4.01 ± 0.67; Depression: Mean 3.94 ± 0.91; Anxiety: Mean 3.75 ± 0.85; Phobic anxiety: Mean 2.74 ± 0.69; Obsession: Mean 2.76 ± 0.59; Interpersonal sensitivity: Mean 2.91 ± 0.84; Hostility: Mean 2.50 ± 0.76; Paranoid ideation: Mean 2.52 ± 0.85 (All significantly higher vs. norm, *p* < 0.01 except Psychoticism).	Disease duration (>10 years): Increased SCL-90 scores for Somatization, Obsession, Interpersonal sensitivity, Depression, Anxiety, Hostility, Phobic anxiety (*p* < 0.05). Pneumoconiosis stage (I to III): Increased SCL-90 scores for Somatization, Obsession, Interpersonal sensitivity, Depression, Anxiety, Hostility, Phobic anxiety (*p* < 0.05).
Chen et al. (34)	Chongqing, China	Cross-sectional (*n* = 212 CWP patients)	SF-36 Domain Scores (vs. controls, *p* < 0.01): Vitality (VT): 79.24 ± 8.93 vs. 88.44 ± 8.25; Role Emotional (RE): 83.26 ± 9.13 vs. 91.62 ± 9.98; Mental Health (MH): 82.99 ± 7.34 vs. 84.75 ± 6.97.	Multivariate Regression (Standardized *β*): Mental Health Composite (VT, SF, RE, MH): Welfare Satisfaction: *β* = 0.474 (*p* < 0.001); Good Healthcare Access: *β* = 0.415 (*p* < 0.001) Self-care Ability: *β* = −0.352 (*p* < 0.001); Health Education: *β* = 0.1458 (*p* = 0.0015).
Chen (44)	Xuzhou, China	Cross-sectional (*n* = 120 coal workers’ pneumoconiosis patients: 60 in the specialized ward vs. 60 in general wards)	Higher SCL-90 scores in the general ward group: total mean score 1.54 ± 0.40 vs. 2.26 ± 0.70; depression score 2.10 ± 0.44 vs. 2.30 ± 0.54; anxiety score 1.35 ± 0.52 vs. 2.27 ± 0.80; significant differences in all factors (*p* < 0.01).	Concurrent infection frequency: higher in the general ward (10.58 ± 1.5 vs. 4.8 ± 0.8 per year, *t* = 8.85, *p* < 0.01); age: older in the specialized ward (75.5 ± 1.2 vs. 62.1 ± 0.3 years, *t* = 10.16, *p* < 0.01).
Chen and Li (27)	Shanghai, China	Cross-sectional (*n* = 100 pneumoconiosis patients)	Self-perceived burden: total score 62.87 ± 11.58; highest in care physical burden dimension (3.09 ± 0.24); depression (PHQ-9) and anxiety (GAD-7) scores positively correlated with burden (*r* = 0.406 and *r* = 0.458, *p* < 0.001).	Multiple regression predictors: medical payment self-payment (*β* = 9.399, *p* < 0.001), rural residence (*β* = 4.550, *p* < 0.001), age ≤60 years (*β* = −3.098, *p* = 0.010), no occupational disease subsidy (*β* = 1.248, *p* = 0.026), higher PHQ-9 score (*β* = 1.207, *p* < 0.001), higher GAD-7 score (*β* = 1.191, *p* = 0.001).
Deng et al. (13)	China	Case–control (*n* = 40 older silicosis-TB patients vs. 40 silicosis-only controls)	Depression: SDS score: 44.8 ± 6.2 in silicosis-TB group vs. 36.9 ± 7.2 in controls (*p* < 0.05); SCL-90 subscores significantly higher in silicosis-TB group (all *p* < 0.05): Depression (2.0 ± 0.7), Somatization (1.9 ± 0.8), Interpersonal sensitivity (1.9 ± 0.6), Anxiety (1.9 ± 0.7), Obsessive-compulsive (1.8 ± 0.6), Hostility (1.8 ± 0.7), Phobic anxiety (1.7 ± 0.4), Paranoid ideation (1.8 ± 0.5), Psychoticism (1.6 ± 0.6).	Significant risk factors for depression in Silicosis-TB patients (Adjusted OR): Worsening depression: OR = 6.31 (95% CI 1.77–29.25); Social dissatisfaction: OR = 2.45 (95% CI 1.01–3.08); Suicidal ideation: OR = 5.10 (95% CI 2.10–6.99); Treatment refusal: OR = 4.34 (95% CI 2.11–4.46).
Fang (23)	China	Cross-sectional (*n* = 35 silicosis with tuberculosis patients vs. 35 silicosis patients)	Depression: Prevalence 48.57% in the silicosis with tuberculosis group vs. 17.14% in the silicosis group, based on HAMD scores ≥8. Mean HAMD score: 15.31 ± 11.44 for silicosis with tuberculosis vs. 8.09 ± 5.56 for silicosis.	Not investigated or reported; the study only compared depression rates/scores between groups without analyzing covariates or adjusted impact factors.
Hou and Li (45)	China	Cross-sectional (*n* = 78 hospitalized coal workers’ pneumoconiosis patients vs. 50 control miners)	Higher SCL-90 scores compared to national norm: somatic (1.72 ± 0.56 vs. 1.37 ± 0.48), obsession (1.92 ± 0.63 vs. 1.62 ± 0.58), interpersonal sensitivity (1.90 ± 0.65 vs. 1.65 ± 0.61), depression (1.88 ± 0.58 vs. 1.50 ± 0.59), anxiety (1.72 ± 0.61 vs. 1.39 ± 0.43), hostility (1.78 ± 0.54 vs. 1.46 ± 0.55), phobia (1.69 ± 0.49 vs. 1.23 ± 0.41), paranoia (1.70 ± 0.55 vs. 1.43 ± 0.57), psychoticism (1.60 ± 0.52 vs. 1.29 ± 0.42), and other (1.67 ± 0.43 vs. 1.31 ± 0.44); all *p* < 0.01.	N/A
Huang et al. (46)	Chongqing, China	Cross-sectional (*n* = 212 coal workers’ pneumoconiosis patients)	Higher SCL-90 scores compared to national norm: somatic (1.68 ± 0.43 vs. 1.37 ± 0.48), obsession (1.92 ± 0.54 vs. 1.62 ± 0.58), depression (1.82 ± 0.62 vs. 1.50 ± 0.59), anxiety (1.88 ± 0.49 vs. 1.39 ± 0.43), phobia (1.81 ± 0.43 vs. 1.23 ± 0.41), paranoia (1.66 ± 0.58 vs. 1.43 ± 0.57), psychoticism (1.40 ± 0.38 vs. 1.29 ± 0.42), and total score (150.92 ± 0.37 vs. 129.60 ± 0.32); no significant difference in interpersonal sensitivity or hostility. All significant differences had *p* < 0.05.	In regression analysis: Self and experience disharmony (*β* = 0.442), objective support (*β* = −0.215), general self-congruency (*β* = 0.194), total social support (*β* = −0.086).
Huang et al. (47)	Shenzhen, China	Cross-sectional (*n* = 196 male CWP patients)	Anxiety (SAS): Mean score 41.98 ± 7.76 (significantly higher than Chinese norm, *p* < 0.01). Prevalence: 59.18% scored ≥50 (36.73% mild, 15.81% moderate, 7.63% severe anxiety). Depression (SDS): Mean score 47.10 ± 9.73 (significantly higher than Chinese norm, *p* < 0.01). Prevalence: 74.50% scored ≥0.5 (28.57% mild, 30.10% moderate, 20.92% severe depression).	Multivariate analysis (SAS): Number of accompanying symptoms (*β* = 1.411, *p* < 0.01), CWP stage (*β* = 2.069, *p* < 0.05). Multivariate analysis (SDS): Age (*β* = 0.210, *p* < 0.01), number of accompanying symptoms (*β* = 1.836, *p* < 0.01), respect level (*β* = −3.960, *p* < 0.01), CWP stage (*β* = 2.080, *p* < 0.05).
Kong et al. (48)	China	Cross-sectional (*n* = 200 CWP patients vs. 100 controls)	Depression (SCL-90 Factor 4): Prevalence 73.0% in CWP patients vs. 44.0% in controls (χ^2^ = 24.144, *p* < 0.01). Mean factor score 2.15 ± 0.29 (CWP) vs.1.85 ± 0.34 (controls) vs. 1.50 ± 0.59 (national norm; *p* < 0.01). Anxiety (SCL-90 Factor 5): Prevalence 52.0% in CWP patients vs. 28.0% in controls (χ^2^ = 15.584, *p* < 0.01). Mean factor score 2.04 ± 0.33 (CWP) vs. 1.85 ± 0.34 (controls) vs. 1.39 ± 0.43 (national norm; *p* < 0.01).	Univariate analysis: CWP patients showed significantly higher depression and anxiety scores than both controls and national norms (*p* < 0.01). No multivariate impact factors reported.
Li et al. (49)	Shandong, China	Cross-sectional (*n* = 526 pneumoconiosis patients)	Mental health dimension (SF-36): Score 61.51 ± 16.64 vs. national norm 68.47 ± 16.90; all SF-36 dimensions lower than national norms.	Dyspnea severity, limited physical activity, economic burden, and self-care ability significantly affected mental health (all *p* < 0.05, based on multiple linear regression).
Lian et al. (8)	China	Cross-sectional (*n* = 120 pneumoconiosis patients vs. 128 controls)	Total SCL-90 score: 148.03 ± 28.95 vs. controls 117.69 ± 15.07; prevalence of psychological problems: 37.5% in patients vs. 10.9% in controls; higher scores in somatization, obsessive-compulsive, depression, and anxiety.	Higher pneumoconiosis stage (stage II: OR = 2.808, 95% CI 1.164–4.813; stage III: OR = 2.028, 95% CI 1.481–3.368), FEV1 (OR = 0.759, 95% CI 0.585–0.984), and higher education level (middle school: OR = 0.292, 95% CI 0.105–0.809; high school or above: OR = 0.329, 95% CI 0.102–0.960) significantly impacted mental health.
Liao (50)	Chongqing, China	Cross-sectional (*n* = 232 silicosis patients vs. 116 controls)	Depression: Prevalence 52.9% (HAMD ≥ threshold for mild/moderate/severe); HAMD mean score 21.1 ± 0.2 vs. 9.2 ± 3.2 in controls (*p* < 0.05). Anxiety: Prevalence 57.8% (HAMA ≥ threshold for marked/anxiety or severe); HAMA mean score 16.1 ± 9.1 vs. 8.2 ± 4.1 in controls (*p* < 0.05).	None reported quantitatively via adjusted OR. Silicosis disease stage (I, II, III) showed no significant association with HAMD/HAMA scores (*p* > 0.05). Discussion mentions fear, economic hardship, job loss, and social status as contributors.
Lin (51)	Fujian, China	Cross-sectional (*n* = 200 silicosis patients)	Depression: SDS mean score 55.10 ± 5.10 (significantly above the Chinese norm cutoff of 41). Anxiety: SAS mean score 49.60 ± 4.20 (significantly above the Chinese norm cutoff of 43).	Diagnosis difficulty and unreimbursed costs: OR = 0.787 (95% CI 0.663–0.942). Perceived incurability of silicosis: OR = 1.527 (95% CI 1.021–2.277). Poverty: OR = 2.123 (95% CI 1.164–3.897). Chronic diseases/complications: OR = 1.635 (95% CI 1.222–2.312).
Liu and Cui (21)	China	Cross-sectional (*n* = 142 coal workers with pneumoconiosis)	Anxiety: Prevalence 61.97% (SAS ≥ 50); Depression: Prevalence 86.62% (SDS ≥ 53).	For anxiety: Comorbidity count: OR = 0.39 (*p* < 0.05); For depression: Age: OR = 0.41 (*p* < 0.05), Disease stage: OR = 0.40 (*p* < 0.05), Comorbidity count: OR = 0.71 (*p* < 0.05).
Liu et al. (38)	Ningxia, China	Cross-sectional (*n* = 530 coal workers with pneumoconiosis)	Depression: Mean score 2.84 ± 0.60 vs. national norm 1.47 ± 0.55 (*p* < 0.05); Anxiety: Mean score 2.63 ± 0.68 vs. national norm 1.40 ± 0.37 (*p* < 0.05).	Subjective support: *r* = −0.848 (*p* < 0.001); Support utilization: *r* = −0.895 (*p* < 0.001); Total social support: *r* = −0.826 (*p* < 0.001).
Luo et al. (28)	27 provinces, China	Cross-sectional (*n* = 951 pneumoconiosis+TB patients)	Pain/Discomfort: 85.4% reported problems (EQ-5D-3L); Anxiety/Depression: 67.8% reported problems (EQ-5D-3L); Life quality: Median utility score 0.562 vs. 0.686 in pneumoconiosis-only (*p* < 0.001); Self-rated health: 53.7 ± 18.4 vs. 59.3 ± 17.7 (*p* < 0.001).	Significant predictors of reduced health utility: Advanced pneumoconiosis stage (*β* = −0.148 for stage III vs. undiagnosed, *p* = 0.002); ≥3 comorbidities (*β* = −0.288, *p* < 0.001); Low personal income <5,500 CNY/year (reference); Acute symptom exacerbation (*β* = −0.153, *p* < 0.001); Lack of social support (*β* = −0.087, *p* = 0.009).
Peng et al. (24)	Shaoguan, China	Cross-sectional (*n* = 66 silicosis+TB patients vs. 64 silicosis controls)	Depression: Prevalence 71.21% (SDS score >53) in silicosis+TB group vs. lower in controls (*p* < 0.05) Anxiety: SCL-90 anxiety subscale score 1.87 ± 0.70 vs. 1.60 ± 0.50 (*p* < 0.05).	Identified risk factors: Depression exacerbation, suicidal ideation, social dissatisfaction, and treatment refusal. The SCL-90 showed significantly higher scores across all domains (somatization, obsession, depression, anxiety, etc.) in silicosis+TB group vs. controls (all *p* < 0.05).
Qin et al. (17)	Guangxi, China	Cross-sectional (*n* = 326 pneumoconiosis patients)	Anxiety symptoms: Prevalence 9.5% (HAMA ≥7); Depression symptoms: Prevalence 16.3% (HAMD ≥8).	Anxiety: Current smoking (OR = 6.498, 95% CI 1.842–22.932); Hypertension comorbidity (OR = 5.306, 95% CI 2.331–12.076); Tuberculosis comorbidity (OR = 3.168, 95% CI 1.327–7.567); Depression: Higher education level (OR = 2.085, 95% CI 1.102–3.943); COPD comorbidity (OR = 2.203, 95% CI 1.134–4.277).
Song et al. (41)	Sichuan, China	Cross-sectional (*n* = 136 pneumoconiosis-tuberculosis patients)	Significantly higher SCL-90 total score (157.72 ± 36.92) vs. Chinese norm (129.96 ± 38.76; *p* < 0.001).	Negative association between social support and psychological symptoms: Total social support score: *β* = −0.348 (*p* < 0.001); Subjective support: *β* = −0.205 (*p* = 0.011); Support utilization: *β* = −0.213 (*p* = 0.015).
Sun et al. (37)	Shenyang, China	Cross-sectional (*n* = 208 pneumoconiosis patients)	Depression: Prevalence 42.8% (GDS > 5); Mean GDS score 5.71	High income: OR = 0.79 (95% CI 0.664–0.941); Longer dust exposure duration: OR = 1.526 (95% CI 1.022–2.278); Higher pneumoconiosis stage (II-III vs. I): OR = 2.129 (95% CI 1.163–3.896)
Wang et al. (53)	China	Cross-sectional (*n* = 200 coal workers’ pneumoconiosis patients vs. 100 controls)	Elevated SCL-90 scores (vs. controls/norms): Somatization, obsessive-compulsive, depression, anxiety, etc.	No adjusted impact factors reported.
Wang and Liu (29)	Tongling, China	Cross-sectional (*n* = 121 silicosis patients)	Anxiety: Prevalence 14.9% (HADS-A > 9).	In Silicosis Patients (Adjusted OR): Severe respiratory symptoms: OR = 5.6 (95% CI 1.1–28); Stage III silicosis: OR = 7.8 (95% CI 1.2–50).
Wang and Gao (54)	Beijing, China	Cross-sectional (*n* = 120 coal workers’ pneumoconiosis patients)	Anxiety: SAS score 43.76 ± 6.79 (vs. Chinese norm 33.80 ± 5.90); Depression: SDS score 48.58 ± 12.79 (vs. Chinese norm 41.88 ± 10.57).	Social support negatively correlated: Anxiety with friend support: *r* = −0.242; Depression with family support: *r* = −0.248.
Wang et al. (25)	Shandong, China	Cross-sectional (*n* = 50 silicosis with tuberculosis patients vs. 50 silicosis patients)	Depression: SDS score 45.9 ± 5.1 in silicosis with TB group vs. 38.0 ± 6.1 in silicosis group; SCL-90 depression dimension score 2.1 ± 0.6 in silicosis with TB group vs. 1.72 ± 0.7 in silicosis group.	In Silicosis Patients with Tuberculosis (Adjusted OR): Refusal of treatment: OR = 4.336 (95% CI 2.105–4.456); Suicide ideation: OR = 5.097 (95% CI 2.102–6.985); Social dissatisfaction: OR = 2.445 (95% CI 1.005–3.079); Depression aggravation: OR = 6.313 (95% CI 1.768–29.248).
Wei and Zhang (39)	China	Cross-sectional (*n* = 200 Coal Workers’ Pneumoconiosis (CWP) patients vs. 100 dust-exposed controls)	SCL-90 Total Symptom Index abnormality rate: 60.0% (120/200) in the CWP group vs. 31.0% (31/100) in controls. By education: Illiterate 69.1% (47/68), Primary 51.5% (48/94), Junior high+ 65.8% (25/38). By stage: Stage I 54.6% (53/97), Stage II 63.8% (60/94), Stage III 77.8% (7/9).	Education level is significantly associated with abnormality rate (χ^2^ = 6.014, *p* < 0.05). The pneumoconiosis stage showed a non-significant trend (χ^2^ = 2.921, *p* > 0.05). (ORs not provided).
Wei and Yu (55)	Yueyang, China	Cross-sectional (*n* = 79 pneumoconiosis patients vs. 79 administrative/logistics controls)	Significantly higher SCL-90 scores (*p* < 0.05) in patients vs. controls: Depression (24.42 ± 4.00), Anxiety (35.00 ± 6.10), Somatization (17.97 ± 5.42), Obsessive-compulsive (27.40 ± 5.69), Hostility (15.26 ± 4.01), Phobic anxiety (15.28 ± 4.07), Paranoid ideation (22.17 ± 4.14), Psychoticism (13.13 ± 6.42).	Higher SCL-90 scores (*p* < 0.05) linked to: Family life (4.02 ± 0.68), Conflicts (2.76 ± 0.60), Treatment burden (3.75 ± 0.87), Value/Respect (3.85 ± 0.88), Interpersonal relationships (2.92 ± 0.83), Economic burden (2.74 ± 0.65), Rights protection (2.49 ± 0.87), Social adaptation (3.87 ± 0.90). (ORs not provided).
Xiang and Liu (35)	Enshi, China	Cross-sectional (*n* = 120 miners with pneumoconiosis)	Mental Health Problems (SCL-90 Positive): 78.33% (94/120). Specific Symptoms (SCL-90 Factor Positive Rate): Somatization: 31.67%, Obsessive-Compulsive: 26.67%, Interpersonal Sensitivity: 26.67%, Anxiety: 19.17%, Depression: 17.50%.	Education Level: Significantly higher mental health problem positive rate in patients with lower education (*p* < 0.05). Coping Style (CSQ Scores in Positive vs. Negative Group): Higher Self-blame (*p* < 0.01), Fantasy (*p* < 0.01), Avoidance (*p* < 0.01), Rationalization (*p* < 0.01); Lower Help-seeking (*p* < 0.05). Correlation (SCL-90 score): Positive correlation with Self-blame (*p* < 0.05); Negative correlation with Help-seeking (*p* < 0.05).
Xie et al. (56)	Hangzhou, China	Cross-sectional (*n* = 63 pneumoconiosis patients vs. 65 dust-exposed controls)	Depression Score (SDS): Pneumoconiosis Group Mean: 54.35 ± 2.47; Control Group Mean: 45.45 ± 3.13 (*p* < 0.05). Within the pneumoconiosis Group, SDS score significantly increased with disease stage (I: 54.22 ± 8.13, II: 55.11 ± 7.45, III: 56.44 ± 6.78, *F* = 3.170, *p* < 0.05; significant differences between all stages). Significantly higher SDS score in patients with ≥3 respiratory symptoms (*p* < 0.05).	Disease Stage: Significantly associated with higher SDS score (OR = 2.549, 95% CI = 1.415–4.519). Number of Respiratory Symptoms: Significantly associated with higher SDS score (OR = 1.037, 95% CI = 1.010–1.012).
Yan et al. (15)	Huaibei, China	Experimental study (*n* = 50 coal workers’ pneumoconiosis patients: 25 intervention vs. 25 control)	Fatigue: Post-intervention score 3.69 ± 0.09 (intervention) vs. 4.71 ± 0.70 (control); Anxiety: Post-intervention score 43.1 ± 7.9 (intervention) vs. 49.6 ± 9.6 (control); Depression: Post-intervention score 45.7 ± 8.3 (intervention) vs. 50.1 ± 8.3 (control); Sleep disturbances: Post-intervention sleep quality improved (17 with no sleep disorder in intervention vs. 2 in control).	Aerobic exercise intervention: Significant reduction in fatigue (*t* = 7.23, *p* < 0.001), anxiety (*t* = 5.07, *p* < 0.001), depression (*t* = 4.43, *p* < 0.001), and sleep disturbances (*p* < 0.05).
Yang (16)	China	Tracking survey (*n* = 57 pneumoconiosis patients)	SAS score: 47.6 ± 3.2 (cutoff >40); SDS score: 54.3 ± 3.8 (cutoff >41).	Clinical stage III vs. I: higher SAS/SDS (*p* < 0.05); ≥3 comorbidities vs. <3: higher SAS/SDS (*p* < 0.05); Lower education (primary school) associated with reduced SAS/SDS scores (*p* < 0.05).
Yang et al. (57)	Chongqing, China	Cross-sectional (*n* = 295 pneumoconiosis patients)	Overall psychological problems: Prevalence 24.07% (SCL-90 ≥ 160); Somatization: 47.80% positive; Sleep and diet problems: 32.20% positive; Obsessive-compulsive symptoms: 24.41% positive; Depression: 18.98% positive; Anxiety: 17.63% positive. SCL-90 scores: Somatization 2.07 ± 0.69, Depression 1.69 ± 0.68, Anxiety 1.62 ± 0.63.	In pneumoconiosis patients (Adjusted OR): Number of hospitalizations due to pneumoconiosis: OR = 1.612 (95% CI 1.266–2.053); Stage of pneumoconiosis: OR = 1.413 (95% CI 1.002–1.994); Age: OR = 0.923 (95% CI 0.886–0.963).
Yao et al. (14)	Shandong, China	Retrospective analysis (*n* = 57 pneumoconiosis patients vs. 724 healthy controls)	Higher SCL-90 total score: 174.61 ± 64.77 vs. 129.96 ± 38.76; Somatization: 2.45 ± 0.86 vs. 1.38 ± 0.49; Depression: 1.98 ± 0.82 vs. 1.51 ± 0.60; Anxiety: 1.97 ± 0.84 vs. 1.41 ± 0.44. Death ideation: 19.3%.	Age: OR = 1.090 (95% CI: 1.004–1.184, *p* < 0.05); Negative Coping: OR = 8,007.941 (95% CI: 60.453–1,060,780.289, *p* < 0.01).
Ye et al. (58)	Guizhou, China	Cross-sectional (*n* = 208 pneumoconiosis patients)	SCL-90 total score: 162.00 ± 64.77 vs. national norm 129.96 ± 38.76, significant increase (*p* < 0.001); Depression factor: Score 1.75 ± 0.75 vs. norm 1.50 ± 0.59, significant increase (*p* < 0.001); Anxiety factor: Score 1.80 ± 0.82 vs. norm 1.39 ± 0.43, significant increase (*p* < 0.001).	Pneumoconiosis stage III vs. stage I: SCL-90 total score higher (170.09 ± 69.06 vs. 155.44 ± 64.77, *p* < 0.05); Social support not significantly correlated with psychological symptoms (all correlation coefficients *p* > 0.05).
Ye et al. (59)	Ma’anshan City, China	Cross-sectional (*n* = 494 pneumoconiosis patients)	Mental health dimension (SF-36 MH): Score 53.34 ± 21.74 vs. national norm 68.47 ± 16.90, significant decline (*p* < 0.001); Vitality dimension (SF-36 VT): Score 42.66 ± 19.02 vs. norm 68.17 ± 17.63, significant decline (*p* < 0.001); Social functioning dimension (SF-36 SF): Score 56.52 ± 28.10 vs. norm 80.67 ± 19.98, significant decline (*p* < 0.001).	For quality of life total score (multivariate linear regression): Age <59 years: *β* = 15.563 (*p* < 0.001); High school education or above: *β* = 5.358 (*p* < 0.05); No chronic disease: *β* = 6.897 (*p* < 0.001); Living with family: *β* = 10.349 (*p* < 0.001); Dust exposure ≤9 years: *β* = 12.386 (*p* < 0.001); Pneumoconiosis stage I: *β* = 15.152 (*p* < 0.001).
Yu et al. (60)	Hunan, China	Cross-sectional (*n* = 613 pneumoconiosis patients)	Anxiety/Depression: Prevalence 21.0% (abnormal on EQ-5D anxiety/depression dimension).	Higher anxiety/depression prevalence in stage II and III patients compared to stage I (*p* < 0.02); higher prevalence in simple pneumoconiosis patients compared to those eligible for lung lavage (*p* < 0.02).
Yue et al. (61)	China	Cross-sectional (*n* = 104 pneumoconiosis patients vs. 35 non-pneumoconiosis COPD controls)	Anxiety: Mean SAS score 45.6–49.2 in pneumoconiosis groups vs. 31.4 in controls; Prevalence of abnormal (SAS > 50) 52.9–53.1% vs. 25.7%. Depression: Mean SDS score 44.3–48.3 vs. 30.8; Prevalence of abnormal (SDS > 50) 50.0–52.9% vs. 22.8%.	Pneumoconiosis patients had significantly higher anxiety and depression scores and prevalence compared to controls (*p* < 0.05).
Zhang et al. (62)	China	Cross-sectional (*n* = 80 male coal workers’ pneumoconiosis (CWP) inpatients vs. *n* = 100 retired miners)	Obsessive-compulsive symptoms (SCL-90): Prevalence 74.25% (Factor score ≥2) in CWP group vs. 42.00% in controls (χ^2^ = 18.19, *p* < 0.01); Mean factor score 2.28 ± 0.38 in CWP vs. 1.98 ± 0.32 in controls (u = 46.89, *p* < 0.01) and vs. Chinese norm 1.62 ± 0.58 (u = 21.25, *p* < 0.01).	Higher obsessive-compulsive symptom prevalence and scores are significantly associated with having CWP (vs. controls). Increased symptom severity was associated with higher pneumoconiosis stage and older age in the CWP group (statistical values not explicitly quantified).
Zhang (42) Zhang et al. (18)	Qinhuangdao, China Xuzhou, China	Cross-sectional (*n* = 120 male migrant pneumoconiosis patients) Cross-sectional (*n* = 133 male pneumoconiosis inpatients)	SCL-90: Somatization: Mean 5.01 ± 0.56; Depression: Mean 4.56 ± 0.68; Anxiety: Mean 4.25 ± 0.35; Hostility: Mean 2.95 ± 0.92; Phobic anxiety: Mean 2.83 ± 0.54; Obsession: Mean 2.93 ± 0.71; Interpersonal sensitivity: Mean 3.02 ± 0.97; Paranoid ideation: Mean 2.75 ± 0.28 (All significantly higher vs. norm, *p* < 0.01 except Psychoticism). Anxiety (SAS): Prevalence 60.90% (SAS standard score ≥50); Mean score 41.99 ± 6.84 (significantly higher than Chinese norm: 29.78 ± 10.07, *t* = 20.605, *p* < 0.001). Depression (SDS): Prevalence 87.22% (SDS index ≥0.5); Mean score 48.98 ± 8.46 (significantly higher than Chinese norm: 33.46 ± 8.55, *t* = 21.156, *p* < 0.001).	Pneumoconiosis stage (I to III): Increased SCL-90 scores for Somatization, Obsession, Interpersonal sensitivity, Depression, Anxiety, Hostility, Phobic anxiety (*p* < 0.01). Migrant worker status. Severe physical symptoms (Somatization mean >4). Multivariate regression (Beta coefficients): Increased anxiety: Number of comorbidities (Beta = 0.507, *p* < 0.001). Increased depression: Pneumoconiosis stage (Beta = 0.150, *p* = 0.017); Age (Beta = 0.231, *p* < 0.001); Number of comorbidities (Beta = 0.574, *p* < 0.001). (*R*^2^ SAS = 0.573, SDS = 0.538).
Zhang et al. (63)	Xuzhou, China	Cross-sectional (*n* = 205 male CWP inpatients)	Depression: 78.5% prevalence (SDS). Anxiety: 59.0% prevalence (SAS).	Physical Domain: Self-care ability (*β* = 0.350, *p* < 0.01), SDS score (*β* = −0.303, *p* < 0.01), Housing satisfaction (*β* = 0.199, *p* < 0.01), Social support (*β* = 0.186, *p* < 0.01). Psychological Domain: Other support (*β* = 0.335, *p* < 0.01), SDS score (*β* = −0.255, *p* < 0.01), Housing satisfaction (*β* = 0.134, *p* = 0.039). Social Domain: Housing satisfaction (*β* = 0.734, *p* < 0.01), Social support (*β* = 0.155, *p* = 0.01). Environmental Domain: SAS score (*β* = −0.344, *p* < 0.01), Self-care ability (*β* = 0.298, *p* < 0.01), Family support (*β* = 0.142, *p* = 0.017).
Zhang and Lu (64)	Xuzhou, China	Cross-sectional (*n* = 205 male CWP inpatients)	Depression: 78.5% prevalence (SDS). Anxiety: 59.0% prevalence (SAS).	SGRQ Correlations: Significant negative correlation between WHOQOL-BREF domain scores (Physical, Psychological, Social, Environmental) and SGRQ dimension scores (Activity, Symptoms, Impacts, Total) (*p* < 0.05 or *p* < 0.01). Significant positive correlation between SAS/SDS scores and SGRQ dimension scores (*p* < 0.01). Higher SGRQ scores (worse function) in patients with anxiety or depression. (*p* < 0.01).
Zhao et al. (33)	Zaozhuang, China	Cross-sectional (*n* = 43 male silicosis inpatients)	Depression: 72.1% prevalence (SDS standard score ≥53). Higher in Stage III (81.8%) vs. Stage I (63.2%).	Patient Concerns (Questionnaire): Disease progression/complications (86.0%), Feeling a burden (69.8%), Reduced social activity (51.2%), Lack of care (44.2%), Financial hardship (34.9%). Associated Factors: Advanced disease stage, long hospitalization (>2 years: 78.3% depression prevalence), Low education/literacy, Severe physical symptoms, Functional impairment.

Before reporting the key results, it is necessary to note that in the reviewed studies, the core concepts were measured in substantially different ways. Assessment tools for depression varied significantly. Studies in English mostly used the Geriatric Depression Scale (GDS, six studies) and the Hospital Anxiety and Depression Scale-Depression subscale (HADS, two studies). Conversely, studies in Chinese most often employed the depression subscales of the Symptom Checklist-90 (SCL-90, 19 studies), the Self-Rating Depression Scale (SDS, 14 studies), and the Hamilton Depression Scale (HAMD, four studies). For anxiety, English studies primarily used the HADS subscale (two studies). Chinese studies mainly used the Self-Rating Anxiety Scale (SAS, 10 studies). Notably, Chinese studies tended to document a wider range of symptoms. They frequently reported multidimensional psychopathology using SCL-90 subscales. For example, 13 studies reported somatization symptoms, and 13 studies reported obsessive-compulsive symptoms. English studies more commonly connected psychological symptoms to specific disease outcomes. These outcomes included Quality of Life (QoL, four studies) or functional impairment, such as activity limitation (three studies).

English-language and Chinese-language studies showed clear differences in research design, suggesting distinct research priorities. The 11 English-language studies (Table 2) included 10 cross-sectional studies and one retrospective cohort study (7). Only the cohort design enabled quantifying depression incidence rates over time (per 1,000 person-years). It allowed calculation of adjusted hazard ratios (aHRs) comparing pneumoconiosis patients with matched controls. This approach directly addressed temporal relationships and quantified risk. The incidence rate reported by Lee et al. (7) indicates an increased risk. However, this study’s cohort design and operational definitions differed significantly from the cross-sectional prevalence studies, contributing to overall heterogeneity. Conversely, among the 44 Chinese language studies (Table 3), 39 employed cross-sectional designs, two employed case–control methodologies (12, 13), 1 employed a retrospective analysis (14), 1 employed an experimental intervention (15), and 1 used a tracking survey (16). The case–control studies specifically enabled direct comparisons. These comparisons examined psychological symptom prevalence and scores between pneumoconiosis patients and non-exposed controls (e.g., depression rates (12)). The experimental study (15) uniquely assessed the effects of a specific intervention (exercise) on symptoms like fatigue and anxiety.

Building on the descriptive findings, a comparative synthesis indicates that differences between the English and Chinese literature go beyond methodological approaches and likely reflect deeper distinctions in research priorities, healthcare systems, and socio-cultural backgrounds. English-language studies predominantly emphasize biological correlates, such as pulmonary function and patient-reported outcomes such as Quality of Life (QoL). Conversely, Chinese-language studies place greater focus on socio-economic mediators, including medical payment burdens, rural residence, and welfare satisfaction. This emphasis may reflect the specific challenges present in China’s social and occupational health environment.

### 3.1 Prevalence of psychological symptoms

Pneumoconiosis patients experience a substantial burden of psychological symptoms, as revealed in both the reviewed English and Chinese studies. This distress typically involves elevated depression and anxiety symptoms. Most of the studies assessed psychological symptoms using validated screening tools and symptom scales. These studies usually reported either the prevalence of clinically significant symptom levels or mean scores. Most of them did not report rates of formal clinical diagnoses based on diagnostic criteria such as the Diagnostic and Statistical Manual of Mental Disorders (DSM) or the International Classification of Diseases (ICD). Therefore, this review is limited to psychological symptoms assessed through screening measures, not clinical diagnosis.

There were substantial differences across the reviewed studies in the reported rates of depression and anxiety among pneumoconiosis patients. These differences may reflect the significant variations in research methods, as discussed earlier. For example, reported prevalence rates may be sensitive to the specific assessment tool used or the diagnostic thresholds set for the tool. For depression, Chinese studies reported prevalence ranging from 16.3% (with HAMD ≥8 (17)) to 87.22% (with SDS index ≥0.5 (18)). English studies reported prevalence ranging from 75.2% (with GDS-15 ≥ 7 (19)) to 86.1% (with HADS-D ≥ 8 (20). For anxiety, Chinese studies reported rates varying from 9.5% (with HAMA ≥7 (17)) to 61.97% (with SAS ≥ 50 (21)). English-language studies reported rates as high as 99.1% (HADS-A ≥ 8 (19)) or 75% (HADS total ≥8 (22)). The heterogeneity in these observations may have reflected the influence of measurement choice, including both the tool and the cutoff criteria used.

Differences in sample characteristics may have further contributed to the observed variations in prevalence. These characteristics included age distribution, such as an older adult focus in Cho and Cho (19) rather than broader age ranges in other studies; specific pneumoconiosis subtypes studied (e.g., Coal Workers’ Pneumoconiosis (CWP), silicosis, silicosis with tuberculosis); and disease severity (e.g., stage I, II, and III). Notably, the reviewed studies differed in their definitions of pneumoconiosis. Some studies focused on the pure forms of the disease, while others included cases with significant comorbidities, such as tuberculosis (13, 23, 24, 25). This complexity makes direct comparisons across studies difficult.

### 3.2 Significant correlates

Both English and Chinese studies identified biological, psychological, and social risk factors. Biological factors included disease severity. Psychological factors included coping styles. Social factors included social support and socioeconomic status. However, the focus on specific factors differed between the studies. English studies uniquely measured the effect of pulmonary function. For example, Tang et al. (26) and Postolache et al. (22) used measures like FEV1% and FVC%. Furthermore, English studies emphasized death anxiety (19). Chinese studies provided more detailed information on socioeconomic mediators, including medical payment burden (27), rural residence (27), and tuberculosis comorbidity, documented in seven studies (including the seven study references, (13, 17, 23, 24, 25, 28, 41)). Chinese studies investigated specific triggers of social dissatisfaction (13, 25).

Biological factors are important to consider. Comorbid conditions such as tuberculosis and chronic obstructive pulmonary disease (COPD) significantly worsened co-occurring depression and anxiety symptoms (18, 25, 28). Disease progression correlated strongly with severity, with advanced pneumoconiosis stages, particularly Stage III (8, 29), showing greater vulnerability (7, 18). More severe dyspnea correlated with worse mental health (22, 30). Reduced lung function (FEV1%/FVC%) is associated with poorer psychological outcomes (26, 31). Greater functional and activity impairment contributed to disease burden (22, 26, 32, 33). Being female or aged ≥80 years further increases depression risk (7).

Psychological factors also played a role. Diminished self-care capacity directly predicted poorer mental health (34), while maladaptive coping strategies (e.g., self-blame) exacerbated symptom severity (35). Existing depression directly intensified symptom burden (30, 36). Patients with low outcome expectations about physical activity showed higher depression severity, while low self-efficacy predicted this (30).

Social factors are important too. These factors included a lack of spouse, religious, or family support (19, 20, 36). Socioeconomic disadvantages, including low education and income, worsened symptoms (7, 19, 37). Patients experiencing high out-of-pocket medical costs (27), rural residency (27), or insufficient social support (38) faced substantially elevated risks. Lower educational attainment was consistently associated with heightened symptom burden (19, 20, 35, 39). Furthermore, longer hospitalization and caregiving burden impaired mental health (19, 36). Protective factors included greater welfare satisfaction, enhanced healthcare access, and financial compensation, which demonstrably mitigated symptom severity (32, 34).

### 3.3 Effectiveness of interventions

Evidence directly showing effective management or intervention strategies is limited to date. This review identified one experimental study. Yan et al. (15) found that recipients of an aerobic exercise intervention showed significant reductions in anxiety and fatigue. This finding suggests that structured physical activity may effectively alleviate these specific symptoms in patients with pneumoconiosis.

The findings reviewed in the previous section regarding biological, psychological, and social factors hint at potential areas for future intervention development. For example, some cross-sectional studies revealed a significant negative link between social support levels and anxiety or depression symptoms (20, 38, 40, 41). Researchers measured social support using tools like the Multidimensional Scale of Perceived Social Support (MSPSS), the Lubben Social Network Scale (LSNS), and the Social Support Rating Scale (SSRS). While this observation does not constitute causal evidence, it suggests that social support may be a valuable resource for treatment, pointing to the potential importance of peer support groups, family counseling, or community programs. Kawaji et al. (30) found lower outcome expectations about physical activity related to worse depression. Self-efficacy played a mediating role, highlighting that promoting positive outcome expectations and self-efficacy could be a reasonable intervention goal. Cognitive-behavioral strategies aimed at changing expectations and reducing unhelpful coping methods, such as self-blame (35), are worth exploring.

Strong cross-sectional links existed between worse physical disease and worse psychological symptoms. These links involve greater disease severity (e.g., higher stage (8, 18, 29), worse lung function (26, 31), more severe breathing problems (21), more other illnesses (e.g., TB, COPD (7, 17, 18, 25, 28, 37, 42)), and increased hospital visits (7). Optimizing medical care for pneumoconiosis and other illnesses is obviously essential. The correlational observation suggests that physical disease management might improve the mental health of pneumoconiosis patients. Higher caregiver depression symptoms were associated with worse patient mental health outcomes (36). This link indicates that reducing caregiver strain could be vital for whole-person care. Evaluating support services, like respite care, deserves attention. Overall, an integrated care model is warranted.

## 4 Discussion

Based on 11 English-language and 44 Chinese-language studies, the review identifies the following key issues.

### 4.1 Key observations

First, psychological symptoms are prevalent among pneumoconiosis patients. However, the prevalence estimates varied substantially across studies, likely due to their methodological differences (e.g., assessment tool and cutoff criteria). Thus, some differences between the studies published in Chinese and those published in English are worth mentioning. For instance, Chinese studies primarily used assessment instruments such as the SCL-90, SDS, and HAMD, whereas English studies relied more on the GDS and HADS. Due to these variations, direct comparisons across studies become difficult. This situation indicates a need for standardization of methods, such as the use of standardized and clinically validated diagnostic tools and criteria.

Second, the analysis regarding factors associated with psychological symptoms revealed some differences between the studies in the two languages. English studies focused more often on biological factors, such as disease severity, lung function decline, and respiratory impairment. Meanwhile, Chinese research was more strongly oriented toward documenting socioeconomic mediators, such as financial burdens from medical expenses and disparities in rural residence. This emphasis in the Chinese studies potentially reflects the predominant concerns facing patients in the country, such as high treatment costs for occupational lung diseases and significant rural–urban health disparities. It may be that, across countries, given their distinct medical systems and social circumstances, the risk factors associated with psychological symptoms among pneumoconiosis patients may vary accordingly. Future studies must consider the context-specificity of the mechanisms underlying such symptoms and of the intervention or management strategies.

Third, there is very limited evidence on how psychological symptoms among pneumoconiosis patients can be effectively managed. Filling this gap would improve care for these patients, but it would require more future studies with an experimental design.

The prominence of socioeconomic factors in Chinese publications likely reflects the specific challenges within China’s healthcare system—for example, high costs for chronic illness management among rural migrants with restricted health insurance. Conversely, the emphasis on biological markers in English-language studies may reflect a stronger orientation toward a biomedical research model in the international literature. These differences suggest that the understanding of psychological distress could be shaped by broader socioeconomic and healthcare contexts.

The objective of our scoping review is to scope the available evidence broadly (65). It has the potential to identify a focused research question for future systematic reviews and meta-analyses (65). Our scoping review identifies a considerable number of quantitative studies on this topic. The results of a scoping review allow us to determine the potential for conducting further reviews and meta-analyses. Based on the evidence mapping, more focused reviews are now appropriate. For example, a systematic review could be performed to synthesize the consistent findings on key correlates of psychological symptoms. A meta-analysis is possible regarding prevalence rates, although such an analysis needs to take into consideration the heterogeneity of assessment instruments and cutoff values used in different studies.

### 4.2 Limitations

The insights discussed above should be considered with the following caveats. First, our bilingual search strategy captured a distinct geographic and epidemiological focus. This is reflected in the significantly higher number of Chinese-language studies (*n* = 44) compared to English-language studies (*n* = 11). As mentioned in the Introduction, China has an extremely high global burden of pneumoconiosis, so it is conceivable that all of the Chinese-language studies focused only on samples from various provinces in mainland China. Conversely, the English-language studies came from more diverse regions, including South Korea, Japan, Romania, Taiwan (China), Hong Kong (China), and mainland China. Still, these studies were predominantly conducted in Asia, with one exception representing Eastern Europe. Overall, this limited geographic scope could substantially constrain the external validity of the conclusions drawn from the existing research.

We excluded qualitative studies. As explained, this decision aligned with the specified objectives of our review, which are to map the prevalence of psychological symptoms and identify biological, psychological, and social factors reported in the empirical literature. Despite this decision, we do recognize the value of qualitative studies for a thick description of the lived experiences of pneumoconiosis patients. We admit that this decision may have compromised the comprehensiveness of the evidence base of our review.

### 4.3 Conclusion

This scoping review established that pneumoconiosis patients face a significant and multifaceted psychological burden. To address this issue, a paradigm shift is needed. Moving forward, priorities must include: (1) the adoption of standardized diagnostic tools in both research and clinical practice to enable reliable monitoring and comparison; (2) the development and evaluation of multidisciplinary intervention models that integrate mental health care into standard pneumoconiosis management; and (3) the implementation of policies that integrate mental health assessment into occupational health care programs to ensure a comprehensive approach to the well-being of this vulnerable population. By mapping the available evidence, this review provides a foundation for future systematic reviews and meta-analyses, enabling the synthesis of correlates and prevalence estimates in high-incidence populations.

## Data Availability

The original contributions presented in the study are included in the article/Supplementary material, further inquiries can be directed to the corresponding author.

## Footnotes

^1^ https://doi.org/10.17605/OSF.IO/4NHG9

## References

[1] LeWine HE. Pneumoconiosis. Harvard Health Publishing, Harvard Medical School. (2023). Available online at: https://www.health.harvard.edu/a_to_z/pneumoconiosis-a-to-z (Accessed July 5, 2025)

[2] Zhang S Xiong J Ruan X Ji C Lu H. Global burden of pneumoconiosis from 1990 to 2021: a comprehensive analysis of incidence, mortality, and socio-demographic inequalities in 204 countries and territories. Front Public Health. (2025) 13:1579851. doi: 10.3389/fpubh.2025.1579851, PMID: 40337738 PMC12055836

[3] Qi XM Luo Y Song MY Liu Y Shu T Pang JL . Pneumoconiosis: current status and future prospects. Chin Med J. (2021) 134:898–907. doi: 10.1097/CM9.0000000000001461, PMID: 33879753 PMC8078400

[4] Huang X Liu W Yao Y Wang D Sun Y Chen W. 30-year trends in the disease burden, incidence, and prevention of pneumoconiosis. China CDC Wkly. (2023) 5:856–60. doi: 10.46234/ccdcw2023.163, PMID: 37814647 PMC10560376

[5] Huang Y Loux T Huang X Feng X. The relationship between chronic diseases and mental health: a cross-sectional study. Ment Health Prev. (2023) 32:200307. doi: 10.1016/j.mhp.2023.200307, PMID: 41181831

[6] Verhaak PFM Heijmans MJWM Peters L Rijken M. Chronic disease and mental disorder. Soc Sci Med. (2005) 60:789–97. doi: 10.1016/j.socscimed.2004.06.012, PMID: 15571896

[7] Lee H-M Liu D-Y Hsu H-L Yu T-L Yu T-S Shen T-C . Risk of depression in patients with pneumoconiosis: a population-based retrospective cohort study. J Affect Disord. (2024) 352:146–52. doi: 10.1016/j.jad.2024.02.057, PMID: 38369263

[8] Lian J Zhang H Lin M Zhou Z Zhou L Guo X . Mental health and its influencing factors in middle-aged and elderly patients with pneumoconiosis. Chin J Behav Med Brain Sci. (2024) 33:352–6. doi: 10.3760/cma.j.cn371468-20230915-00111, [In Chinese]

[9] Dai M. Guojia weijian wei chenfei bing zhan zhiye bing zongshu de 90% zhenduan zhouqi guochang qie nan quzheng [National Health Commission: pneumoconiosis accounts for 90% of occupational diseases, with a long diagnosis cycle and difficulty in obtaining evidence]. (2024). Available online at: https://news.cctv.com/2024/04/25/ARTI9SRdjhevVa7j9S5mTijT240425.shtml (Accessed July 5, 2025)

[10] Li J Yin P Wang H Wang L You J Liu J . The burden of pneumoconiosis in China: an analysis from the global burden of disease study 2019. BMC Public Health. (2022) 22:1114. doi: 10.1186/s12889-022-13541-x, PMID: 35659279 PMC9166455

[11] Tricco AC Lillie E Zarin W O'Brien KK Colquhoun H Levac D . PRISMA extension for scoping reviews (PRISMA-ScR): checklist and explanation. Ann Intern Med. (2018) 169:467–73. doi: 10.7326/M18-0850, PMID: 30178033

[12] Cao Z Wang H Yang G Zhang X. Analysis of depression and anxiety symptoms in 160 cases of coal workers' pneumoconiosis. J Ningxia Med Univ. (2013) 35:565–6. doi: 10.16050/j.cnki.issn1674-6309.2013.05.003

[13] Deng J Liang Y Liu C Jin M Xiong Y Wang S. Laonian xifei bing hebing fei jiehe huanzhe yiyu de weixian yinsu ji zhendui xing zhiliao fenxi [analysis of risk factors for depression and personalized interventions in geriatric silicosis patients complicated with tuberculosis]. Hebei Med J. (2016) 38:995–7. doi: 10.3969/j.issn.1002-7386.2016.07.009

[14] Yao L Liu Y He J. Analysis on mental health status and influencing factors of pneumoconiosis patients. Occup Health. (2024) 40:577–80. doi: 10.13329/j.cnki.zyyjk.2024.0136

[15] Yan J Hu N Wang J Zhang G Jiang B Wang K . Influence of aerobic exercise on quality of life of patients with coal labor pneumoconiosis. Chin Nurs Res. (2013) 27:268–70. doi: 10.3969/j.issn.1009-6493.2013.03.047

[16] Yang Z. Factors affecting the survey of anxiety and depression in pneumoconiosis patients. Chin J Clin Ration Drug Use. (2014) 7:100–1. doi: 10.15887/j.cnki.13-1389/r.2014.09.003

[17] Qin Y He F Li J Yang R Liang S. Analysis of anxiety and depression and influencing factors of pneumoconiosis patients in Guangxi from 2022 to 2023. Chin Youjiang Med J. (2024) 52:623–8. doi: 10.3969/j.issn.1003-1383.2024.07.008

[18] Zhang M Tang L Shao L Yan X Li J. Investigation on anxiety and depression of pneumoconiosis patients and related influencing factors. Chin Occup Med. (2013) 40:52–4. doi: 10.11763/j.issn.2095-2619.2013.01.020

[19] Cho S Cho O-H. Depression and quality of life in older adults with pneumoconiosis: the mediating role of death anxiety. Geriatr Nurs. (2022) 44:215–20. doi: 10.1016/j.gerinurse.2022.02.018, PMID: 35231755

[20] Han B Yan B Zhang J Zhao N Sun J Li C . The influence of the social support on symptoms of anxiety and depression among patients with silicosis. Sci World J. (2014) 2014:1–6. doi: 10.1155/2014/724804, PMID: 24892079 PMC4032658

[21] Liu Y Cui L. Meikuang gongren chenfei bing huanzhe jiaolv yiyu zhuangkuang jiqi yingxiang yinsu [survey of depression and anxiety symptoms and their influencing factors in coal workers' pneumoconiosis patients]. China Pract Med. (2015) 10:284–5. doi: 10.14163/j.cnki.11-5547/r.2015.21.207

[22] Postolache PA Moscalu M Croitoru A Jimborean G. Functional and nonfunctional parameters in patients with occupational lung diseases. Mater Plast. (2017) 54:535–8. doi: 10.37358/MP.17.3.4889

[23] Fang C. Xifei fei jiehe huanzhe yiyu zhengzhuang qingkuang diaocha ji huli duice [survey of depressive symptoms and nursing interventions in silico-tuberculosis patients]. Int J Nurs. (2014) 33:57–8. doi: 10.3760/cma.j.issn.1673-4351.2014.01.026

[24] Peng Z Ling Y Jian L Lu S. Elderly patients with silicosis with pulmonary tuberculosis risk factors for depression in clinical analysis is extremely. Med J Chin Peoples Health. (2011) 23:1913–4. doi: 10.3969/j.issn.1672-0369.2011.15.044

[25] Wang F Li H Li C. The risk factors of depression in elderly patients with silicosis complicated by pulmonary tuberculosis and targeted countermeasures. Hebei Yi Xue. (2019) 41:929–32. doi: 10.3969/j.issn.1002-7386.2019.06.034

[26] Tang WK Lum CM Ungvari GS Chiu HFK. Health-related quality of life in community-dwelling men with pneumoconiosis. Respiration. (2006) 73:203–8. doi: 10.1159/000088681, PMID: 16195660

[27] Chen G Li Y. The self-perceived burdens of pneumoconiosis patients and the influencing factors. Modern Clin Nurs. (2019) 18:15–20. doi: 10.3969/j.issn.1671-8283.2019.12.003

[28] Luo F Wang H Li T. EQ-5D-3L based quality of life for patients of pneumoconiosis combined with tuberculosis and its influencing factors. Chin J Ind Hyg Occup Dis. (2024) 42:433–41. doi: 10.3760/cma.j.cn121094-20231121-00122 38964907

[29] Wang C Liu L. Tongkuang laonian xifei huanzhe de jiaolv zhuangtai ji yingxiang yinsu [analysis of anxiety status and its influencing factors in geriatric silicosis patients among copper miners]. J Clin Pulmonary Med. (2011) 16:1951–2.

[30] Kawaji T Hasegawa T Uchiyama Y. Dyspnea and outcome expectations are associated with physical activity in persons with pneumoconiosis: a cross-sectional study. BMC Pulm Med. (2022) 22:335. doi: 10.1186/s12890-022-02128-2, PMID: 36056341 PMC9440502

[31] Wang C Yang LS Shi XH Yang YF Liu K Liu RY. Depressive symptoms in aged Chinese patients with silicosis. Aging Ment Health. (2008) 12:343–8. doi: 10.1080/13607860802120938, PMID: 18728947

[32] Liu H Yan B Han B Sun J Yang Y Chen J. Determination of ameliorable health impairment influencing health-related quality of life among patients with silicosis in China: a cross-sectional study. J Int Med Res. (2011) 39:1448–55. doi: 10.1177/147323001103900433, PMID: 21986147

[33] Zhao J Chen J Zhu H Han Q. Xifei huanzhe yiyu zhuangkuang diaocha ji huli [analysis of depression status and nursing interventions in silicosis patients]. J North China Coal Med College. (2003) 5:224–5. doi: 10.19539/j.cnki.2095-2694.2003.02.102

[34] Chen F Zhong M Zhang H. Study on influencing factors of quality of life of coal workers pneumoconiosis patients. Chongqing Med J. (2011) 40:1468–70. doi: 10.3969/j.issn.1671-8348.2011.15.005

[35] Xiang K Liu X. Relationship between negative psychological characteristics and coping styles among patients with miners' pneumoconiosis in Enshi Tujia and Miao autonomous prefecture central hospital. Occup Health (Lond). (2021) 37:145–51. doi: 10.13329/j.cnki.zyyjk.2021.0032

[36] Tang W-K Yip W-C Lum C-M Xiang Y-T Lee E Ungvari GS. Caregiving burden and quality of life of pneumoconiosis caregivers in Hong Kong. Heart Lung. (2011) 40:412–9. doi: 10.1016/j.hrtlng.2010.04.011, PMID: 20630592

[37] Sun J Han B Yan B Liu H. Chenfei huanzhe yiyu zhuangkuang he yingxiang yinsu fenxi [analysis of depressive symptoms and their influencing factors in pneumoconiosis patients]. Natl Med Front China. (2011) 6:94–5. doi: 10.3969/j.issn.1673-5552.2011.21.0060

[38] Liu X Xuan J Fang X Tan Q Ma Y Chang X. Investigation of mental health and social support on patients with coal worker pneumoconiosis and its correlation. Chin Foreign Med Res. (2022) 20:91–4. doi: 10.14033/j.cnki.cfmr.2022.29.024

[39] Wei X Zhang J. Effect of different levels of education and different stages of pneumoconiosis on mental health of coal workers with pneumoconiosis. Occup Health. (2009) 25:354–5. doi: 10.13329/j.cnki.zyyjk.2009.04.052354

[40] Tang WK Lum CM Ng KY Ungvari GS Chiu HFK. Prevalence and correlates of depression in Chinese elderly patients with pneumoconiosis. Aging Ment Health. (2006) 10:177–81. doi: 10.1080/13607860500310310, PMID: 16517493

[41] Song Y Zhang J Chen C Li X Du J Li Z . Chenfei hebing fei jiehe huanzhe xinli jiankang yu shehui zhichi xiangguan xing fenxi [correlation between mental health and social support in pneumoconiosis patients with pulmonary tuberculosis]. Chin J Lung Dis (Electron Edn). (2019) 12:230–2. doi: 10.3877/cma.j.issn.1674-6902.2019.02.026

[42] Zhang G. Mental health status of 120 migrant workers with pneumoconiosis. Occup Health (Lond). (2011) 27:150–1. doi: 10.13329/j.cnki.zyyjk.2011.02.047150

[43] Chen R He L Liu X. 196 Ming chenfei huanzhe xinli jiankang zhuangkuang diaocha fenxi [survey analysis of psychological health status among 196 pneumoconiosis patients]. J Med Forum. (2006) 27:59–60.

[44] Chen L. Butong yiliao huanjing xia meigong chenfei huanzhe xinli yinsu de yanjiu [study on psychological factors in coal workers' pneumoconiosis patients across different healthcare settings]. Jilin Med J. (2012) 33:7698–9.

[45] Hou G Li J. Meigong chenfei bingren xinli jiankang zhuangkuang diaocha [survey of mental health status in coal workers' pneumoconiosis patients]. J Binzhou Med Coll. (2003) 26:306.

[46] Huang J Yi J Chen F Zhang H. Association of mental health with self congruency and social support in coal workers’ pneumoconiosis patients. Chin J Public Health. (2012) 28:668–9.

[47] Huang Z Tang L Ju Q Zhang M. Anxiety and depression in coal worker’s pneumoconiosis patients and its influencing factors. Occup Health. (2014) 30:602–7.

[48] Kong X Wei X Zhang J Sun Z Dong N Yan B. Analysis of the symptoms of depression and anxiety among the coal miners pneumoconiosis patients. Occup and Health. (2008) 24:718–20. doi: 10.13329/j.cnki.zyyjk.2008.08.046718

[49] Li X Ren L Sun F Song Y Gao P. Influencing factors of SF-36 scale on analyzing quality of life in patients with pneumoconiosis. Occup Health. (2023) 39:3169–73. doi: 10.13329/j.cnki.zyyjk.20230817.001

[50] Liao J. Clinical analysis on anxiety and depression of the silicosis patients in mining area. J Modern Med Health. (2006) 22:3412–3.

[51] Lin S. Chenfei bing huanzhe xinli zhuangkuang diaocha ji yingxiang yinsu fenxi [survey and analysis of influencing factors on mental health status in pneumoconiosis patients]. J Med Theory Pract. (2019) 32:3904–6. doi: 10.19381/j.issn.1001-7585.2019.23.074

[52] Tang WK Lum CM Ungvari GS Chiu HFK. P.2.A.002 a geriatric depression scale detects depression in Hong Kong Chinese elderly patients with pneumoconiosis? Eur Neuropsychopharmacol. (2006) 16:S283–4. doi: 10.1016/S0924-977X(06)70282-6

[53] Wang L Zhang J Kong X Wei X Dong N. Research on the psychological health status of coal workers with pneumoconiosis. Occup Health. (2009) 25:1594–5.

[54] Wang Q Gao Y. Meigong chenfei huanzhe jiaolv yiyu yu shehui zhichi de xiangguan xing yanjiu [correlation study between depression and anxiety symptoms with social support in coal workers' pneumoconiosis patients]. Today Nurse. (2015) 6:120–1.

[55] Wei X Yu Y. Investigation and analysis of psychological stress in patients with pneumoconiosis. China Health Stand Manag. (2020) 11:8–10. doi: 10.3969/j.issn.1674-9316.2020.19.004

[56] Xie Q Cao C Wang Y Chu Y Cao R Hu X. Self rating for depression and its influencing factors of patients with pneumoconiosis. Chin Prev Med. (2015) 16:785–8. doi: 10.16506/j.1009-6639.2015.10.008

[57] Yang W Gao L Gao Y Zhang X Luo D. Investigation on mental health status of pneumoconiosis patients in Chongqing based on SCL-90. Occup Health (Lond). (2022) 38:1611–4. doi: 10.13329/j.cnki.zyyjk.2022.0365

[58] Ye X Chen Y Li L Wu L Yuan M. The investigation of psychological and social support for pneumoconiosis patients. J Occup Health Damage. (2016) 31:204–9.

[59] Ye M Gao F Qiu G Gan J Zhou Y Peng B. Study on quality of life and influencing factors of pneumoconiosis patients in Ma’ Anshan City. Occup Health Emerg Rescue. (2024) 42:464–9. doi: 10.16369/j.oher.issn.1007-1326.2024.04.008

[60] Yu Z Liu X Yu D Yang L Xiao X Zhan Z . Analysis on health-related quality of life of migrant workers with pneumoconiosis in Hunan Province. Chin Occup Med. (2023) 50:301–4. doi: 10.20001/j.issn.2095-2619.20230611

[61] Yue J Sun X Song Y. Chenfei bing huanzhe xiaoji qingxu diaocha fenxi [analysis of negative emotions survey in pneumoconiosis patients]. Occup Health. (2005) 21:31–2. doi: 10.13329/j.cnki.zyyjk.2005.01.025

[62] Zhang J Dong N Wei X Sun Z Yan B. Analysis of the compulsive symptoms of the coal workers” pneumoconiosis patients. Occup Health (Lond). (2008) 24:2510–2. doi: 10.13329/j.cnki.zyyjk.2008.23.0502510

[63] Zhang M Ju Q Lu W. Investigation on the quality of life of coal-worker’s pneumoconiosis patients and its influencing factors. Occup Health. (2014) 30:1767–75. doi: 10.13329/j.cnki.zyyjk.2014.13.026

[64] Zhang M Lu W. Study on assessment of quality of life in patients with coal-worker's pneumoconiosis by QOL-BREF and SGRQ. Occup Health (Lond). (2015) 31:3073–6. doi: 10.13329/j.cnki.zyyjk.2015.1060

[65] Munn Z Peters MDJ Stern C Tufanaru C McArthur A Aromataris E. Systematic review or scoping review? Guidance for authors when choosing between a systematic or scoping review approach. BMC Med Res Methodol. (2018) 18:143. doi: 10.1186/s12874-018-0611-x, PMID: 30453902 PMC6245623

