# Supplementary material for: Psychological symptoms and their correlates in pneumoconiosis patients: a bilingual scoping review
Source: Front Public Health. 2025 Nov 13;13:1703091. doi: 10.3389/fpubh.2025.1703091 (PMC12658332; doi:10.3389/fpubh.2025.1703091)
Supplement: Supplementary file 1 [file Data_Sheet_1.docx]

**Any articles published after our searches will not be included in the article and search results.**

PubMed:

("pneumoconiosis"[Mesh] OR "pneumoconiosis") AND ("anxiety"[Mesh] OR "depression"[Mesh] OR "mental health"[Mesh] OR "psychological distress")

Filters applied: English, Humans, from 2000/1/1 - 2025/6/30.

ProQuest:

(SU(pneumoconiosis) OR TIAB(pneumoconiosis)) AND ((SU(anxiety) OR TIAB(anxiety)) OR (SU(depression) OR TIAB(depression)) OR (SU("mental health") OR TIAB("mental health")) OR TIAB("psychological distress"))

ScienceDirect:

pneumoconiosis AND ("mental health" OR anxiety OR depression OR "psychological distress")

Filters applied: English, 2000 – 2025.

APA databases (APA PsycArticles & PsycInfo​)

( (DE="pneumoconiosis" OR TIAB="pneumoconiosis") ) AND

( DE="anxiety" OR TIAB="anxiety" OR

DE="depression (emotion)" OR TIAB="depression" OR

DE="mental health" OR TIAB="mental health" OR

TIAB="psychological distress")

Filters applied: English, 2000/1/1 – 2025/7/5, Male, Female, Peer-reviewed

Scopus:

( TITLE-ABS-KEY ( "pneumoconiosis" ) ) AND ( TITLE-ABS-KEY ( "anxiety" ) OR TITLE-ABS-KEY ( "depression" ) OR TITLE-ABS-KEY ( "mental health" ) OR TITLE-ABS-KEY ( "psychological distress" ) ) AND PUBYEAR > 1999 AND PUBYEAR < 2026 AND ( LIMIT-TO ( LANGUAGE , "English" ) ) AND ( LIMIT-TO ( EXACTKEYWORD , "Human" ) )

Web of Science:

( TS=("pneumoconiosis") ) AND ( TS=("anxiety") OR TS=("depression") OR TS=("mental health") OR TS=("psychological distress") )

Filters applied: English, NOT Publication Years: 1979.

CNKI

(SU=('尘肺病' + '矽肺' + '煤工尘肺') AND SU=('焦虑' + '抑郁' + '心理健康')) OR (SU=('尘肺病' * '心理困扰') OR SU=('矽肺' * '心理应激') OR SU=('煤工尘肺' * '心理痛苦'))

Filters applied: Journal articles, 2000-2025

CBM

("尘肺病"[中文标题:智能] OR "尘肺病"[主题词] OR "尘肺病"[关键词] OR "矽肺"[中文标题:智能] OR "矽肺"[主题词] OR "煤工尘肺"[中文标题:智能]) AND ("焦虑"[主题词] OR "抑郁"[主题词] OR "焦虑"[中文标题:智能] OR "抑郁症"[中文标题:智能] OR "抑郁"[关键词] OR "心理健康"[主题词] OR "心理困扰"[中文标题:智能] OR "心理应激"[关键词])

Filters applied: 2000-2025, Human

Wanfang

(Title:("尘肺病" OR "矽肺" OR "煤工尘肺") OR Keywords:("尘肺病" OR "矽肺" OR "煤工尘肺")) AND (Subject:("焦虑" OR "抑郁" OR "心理健康" OR "心理困扰" OR "心理应激") OR Title:("焦虑" OR "抑郁症" OR "心理健康" OR "心理应激"))

Filters applied: 2000.1.1-2025.7.5, Journal articles

VIP

(T=尘肺病 OR T=矽肺 OR T=煤工尘肺 OR K=尘肺病 OR K=矽肺 OR K=煤工尘肺) AND (R=焦虑 OR R=抑郁 OR R=心理健康 OR R=心理困扰 OR R=心理应激 OR T=焦虑 OR T=抑郁症)

Filters applied: 2000-2025
